# Relationship of Plasma Adiponectin Levels with Acute Coronary Syndromes and Coronary Lesion Severity in North Indian Population

**DOI:** 10.1155/2013/854815

**Published:** 2013-12-10

**Authors:** Amit Mittal, Mohit D. Gupta, Girish Meennahalli Palleda, Aniruddha Vyas, Sanjay Tyagi

**Affiliations:** Department of Cardiology, GB Pant Hospital and Associated Maulana Azad Medical College, Jawahar Lal Nehru Marg, New Delhi 110002, India

## Abstract

Adiponectin is an adipocyte specific cytokine which, in contrast to other adipokines, has been described to have antiinflammatory, antithrombotic, and anti-atherogenic properties. This study evaluates the association between plasma adiponectin levels with acute coronary syndrome (ACS) and angiographic coronary lesion severity in Indian population. Ninety patients included in the study were divided in two groups in 1 : 1 ratio—patients admitted with a diagnosis of ACS and those without ACS. Adiponectin and other risk markers are measured in forty-five consecutive patients in each group undergoing coronary angiography. Patients without ACS were found to have higher adiponectin (16.47 ± 7.88 **μ**g/mL) levels than patients with ACS (9.03 ± 3.13 **μ**g/mL) (*P* < 0.001). In multiple regression analysis adjusted for all other risk markers, higher adiponectin levels remain positively associated with a lower risk of ACS (*P* value > 0.002). The greatest increase in risk for ACS was seen at adiponectin levels ≤12.20 **μ**g/mL in study subjects. The adiponectin levels were inversely related to the angiographic severity of coronary artery stenosis increases (*P* value > 0.02). The study concluded that higher adiponectin levels are independently associated with lower risk of ACS, and patients with severe angiographic coronary artery disease have lower levels of adiponectin.

## 1. Introduction

Obesity is pandemic in industrialized and south Asian countries and has been implicated as a major cause of cardiovascular morbidity and mortality [1–4]. Adipose tissue may play an important role in mediating the chronic inflammatory process implicated in atherosclerosis and coronary artery disease. Adipocyte has an active endocrine function, as it produces several cytokines: interleukin-6 (IL-6), tumour necrosis factor-*α* (TNF-*α*), and adiponectin that is a 30 kDa adipocyte complement-related protein. These cytokines may directly contribute to the development of obesity-related diseases, such as diabetes mellitus, dyslipidemia, hypertension, and atherosclerotic vascular disease [5, 6].

Adiponectin is an adipocyte specific cytokine which, in contrast to other adipokines, has been described to have antiinflammatory, antithrombotic, and anti-atherogenic properties [7–10]. It is abundant in the plasma of normal subjects, but it decreased in conditions such as obesity [11] and type-2 diabetes mellitus [12]. In healthy individuals, low plasma adiponectin levels have been associated with increased risk of cardiovascular events [13]. Therefore, it is suggested that low adiponectin levels may contribute to coronary plaque vulnerability. Furthermore, hypoadiponectinemia has been closely linked to endothelial dysfunction and may be involved in lipid metabolism. Whether adiponectin is an independent risk factor in this signaling network or is mainly associated with other inflammatory proteins such as IL-6 or C-reactive protein or other cardiovascular risk factors is yet unclear. The further elucidation of the pathogenetic role of adiponectin may allow identifying subjects at increased risk for coronary heart disease (CHD), and subsequently leading to more focused preventive measures and eventually to a specific treatment (e.g., application of recombinant adiponectin). Present study aimed to evaluate association between plasma adiponectin levels and acute coronary syndrome in north Indian population.

## 2. Methods

### 2.1. Study Design

The study group consisted of 90 patients of coronary artery disease, and these patients were divided into two subgroups—with (*n* = 45) and without ACS (*n* = 45). All 90 patients who agreed to participate in the study were enrolled and all patients were subjected to coronary angiography for clinical indications (mainly chest pain, dyspnea on exertion, or positive stress test). Patients in ACS subgroup had a final diagnosis of either unstable angina or acute MI, based on clinical feature, biochemical markers, and electrocardiographic changes. Patients were classified as having unstable angina if they had chest pain that was new in onset or if they had a significant unexplained change in the pattern of stable angina (such as increased frequency, increased intensity, increased duration, or decreased response to nitrates) in the previous 2 months. Patients were defined as having an acute MI if they had cardiac marker elevation in association with chest pain or ischemic electrocardiography changes. The exclusion criteria were diabetes mellitus, a smoking history ≥50 pack-years, history of previous coronary revascularization, renal failure, prior chest radiation therapy, and pregnancy. Since this study was designed to look for newer risk factors in Indian subjects, so it was decided to exclude those known risk factors very strongly associated with ACS risk and increased coronary lesion severity. All the recruited patients were subjected to coronary angiography using the standard protocols. Patient was considered to have significant coronary artery disease if the diameter stenosis is >70% by visual estimate. The study population was accordingly divided into normal/single vessel disease/double vessel disease/triple vessel disease (N/SVD/DVD/TVD) based on coronary angiography.

### 2.2. Blood Sampling

Fasting morning blood sample was obtained from all patients enrolled in the study. Blood was immediately placed into the vacutainer tubes, centrifuged, and stored at −70°C until analysis at a later late. Adiponectin was measured by ELISA (BIOVENDOR, USA); leptin was measured by ELISA (DRG, USA), and high sensitivity C-reactive protein was measured by ELISA (CAL BIOTECH, USA).

### 2.3. Statistical Analysis

The collected data was analyzed using SPSS software. Variables were analysized as mean ± SD. Student's *t*-test and wilcoxon ranks-sum test were used to look for significant difference in variables between two groups. Spearman's correlation coefficients were used to find the correlation between adiponectin and other variables. Multiple logistic regression analysis was done to look for the independent association between adiponectin and ACS adjusted for age, gender, BMI, C-reactive protein, LDL/HDL cholesterol ratio, triglycerides, insulin levels, leptin levels, smoking status, and prior MI. Further, the correlation coefficient was calculated to look for association between plasma adiponectin levels and severity of coronary artery stenosis.

## 3. Results

### 3.1. Baseline Characteristics

The baseline demographic and clinical characteristics of two groups are shown in Table 1. Although age and sex are well matched in two groups, more subjects with ACS were smoker, as well as being hypertensive and having higher BMI and family history of premature CAD. The biochemical variables of two groups are shown in Table 2. Leptin levels, hs-CRP, and LDL/HDL ratio were significantly higher in patients with ACS. Adiponectin levels were lower in those with ACS. Subjects with ACS had more cardiovascular risk factors compared to patients without ACS. The angiographic profile of two groups is shown in Table 3. There was no significant difference in two groups with respect to significant CAD (*P*  value = 0.56). Majority of patients without ACS had single vessel disease, while those with ACS majority had triple vessel disease.

### 3.2. Correlations of Adiponectin

Significant associations were seen between adiponectin and several other variables. Adiponectin levels were positively correlated with HDL cholesterol level (*P*  value = 0.002) and negatively correlated with LDL/HDL ratio and hs-CRP level (*P*  value = 0.002 and 0.001) as shown in Table 4. With all other risk factors or biochemical parameter, correlation with adiponectin was nonsignificant.

### 3.3. Association of Adiponectin with ACS

The ACS and non-ACS groups were compared in order to assess for any independent association between adiponectin and the risk of ACS. In multiple regression models adjusted for all the covariates, higher adiponectin levels remained positively associated with a lower risk of ACS (*P*  value = 0.002). The protective association of adiponectin was independent of all the other risk factors, including BMI, CRP, dyslipidemia, and smoking. Utilizing the ROC curve for values of adiponectin and risk of ACS, it was seen that the greatest increase in risk for ACS was seen at adiponectin levels <12.20 *μ*g/mL.

### 3.4. Angiographic Severity of Coronary Artery Stenosis and Adiponectin Levels

Adiponectin levels shows a significantly progressive decline as the angiographic severity of coronary artery stenosis increases (*P* value = 0.023) as shown in Table 5.

## 4. Discussion

### 4.1. ACS and Non-ACS Subjects

In the present study, there were significant differences between ACS and non-ACS subjects with regard to conventional CAD risk factors like BMI, smoking pattern, dyslipidemia, family history of CAD, and C-reactive protein. BMI was significantly higher in ACS group, suggesting the role of obesity not only in causation of atherosclerosis but also in the progression of the disease and even the role in plaque vulnerability. There were more smokers and dyslipidemic subjects in ACS group.

C-reactive protein was significantly higher in patients with ACS, and adiponectin was significantly lower in the ACS subjects, suggesting its protective properties.

### 4.2. Protective Association of Adiponectin with ACS

Adiponectin is a novel adipokine specifically expressed in the adipose tissue and paradoxically lower in obesity [14]. The novel and important finding of the present study is that, using multiple logistic regression model, adiponectin was found to be positively associated with a lower risk of ACS (i.e., high adiponectin level decreased risk of ACS).

Plasma concentrations of adiponectin were significantly lower in patients with ACS than in non-ACS (ACS: 9.03 ± 3.13 *μ*g/mL and non-ACS: 16.47 ± 7.88 *μ*g/mL), thus suggesting that adiponectin may have antiatherosclerotic and antiinflammatory properties and the inflammatory process may be accelerated in patients with low plasma concentrations of adiponectin. Similar observations were suggested in other studies that suggested that measurement of plasma concentrations of adiponectin may be of use for assessing the risk of CAD and may be related to the development of ACS, and higher plasma adiponectin levels are independently associated with a lower risk of ACS, and the greatest increase in risk for ACS in their study was seen at adiponectin levels <5.5 *μ*g/mL [15, 16]. In the present study the protective association of adiponectin was independent of several other recognized cardiovascular risk factors including BMI, smoking, dyslipidemia, and C-reactive protein, and the greatest increase in risk for ACS in the present study was seen at adiponectin levels <12.20 *μ*g/mL. This suggests that the pathophysiological role of adiponectin is related more to the stability of atherosclerotic plaque, although a role for adiponectin in the development of atherosclerotic plaque is also likely. These data suggest that low plasma concentrations of adiponectin are a risk factor for ACS and an independent variable for ACS. Consequently, it is conceivable that low plasma concentrations of adiponectin may facilitate rupture of the atherosclerotic plaques, leading to ACS.

### 4.3. Protective Association between Adiponectin and Coronary Artery Stenosis

In the present study, adiponectin levels decrease as the number of significantly narrowed coronary arteries increases. Similar observation was made by number of previous studies [17, 18] that found that adiponectin levels decreased as a function of number of significantly narrowed coronary arteries and that, in patients with ACS, those with multiple complex lesions had significantly lower adiponectin than those with a single complex lesion. So the present study reinforce, the protective role of adiponectin in the development of atherosclerotic plaque, and also higher adiponectin levels are associated with lower atherosclerotic burden as in the previous studies.

### 4.4. Correlation between Adiponectin and Other CAD Risk Factors

Although the physiological role of adiponectin has not yet been fully elucidated, it may well be involved in the regulation of many of the inflammatory processes or in the lipid metabolisms, which are contributing to atherosclerosis. Adiponectin serum concentrations were strongly correlated with various lipoproteins; among them the strongest correlation was seen with HDL-C and LDL/HDL ratio indicating that there is a close association between adiponectin and HDL metabolism. Present study also found a strong correlation between adiponectin serum concentrations and C-reactive protein. Among the many risk factors involved in atherogenesis, disorders of lipoprotein metabolism are considered to play the most important role. In particular, high LDL and low HDL concentrations are main determinants of CHD risk. We found the strongest correlations of adiponectin serum concentrations with HDL levels. The biochemical mechanisms linking adiponectin to HDL metabolism have not been clarified so far. Adiponectin also seems to have a key role in the metabolic syndrome [19] and may therefore represent the link between obesity (or even more important visceral fat accumulation), insulin resistance, and diabetes. Altogether, this raises the possibility that low adiponectin concentrations may cause low HDL-C and that the pro-atherogenic effects of low adiponectin may be mediated by its effects on HDL metabolism.

### 4.5. Mechanism of the Protective Association of Adiponectin

The exact mechanism of the protective association of adiponectin cannot be established on the basis of the present study; several possibilities should be considered. On the basis of these multiple mechanisms of action, it is likely that the protective effects of adiponectin for ACS are multifactorial. Adiponectin adheres to injured vascular wall, and the antiinflammatory, antiproliferative, and antiapoptotic effects of adiponectin may conceivably lead to greater plaque stability [13, 16, 18, 19]. Adiponectin modifies several recognized cardiovascular risk factors as adiponectin levels are negatively correlated with LDL/HDL ratio and positively correlated with HDL. In addition, adiponectin may be protective against ACS after plaque ruptures through its antithrombotic effects.

### 4.6. Study Limitations

The potential limitations of the present study are the small number of patients' studies. Therefore, the results may not be representative of the population at large. However, they do suggest that further larger studies are warranted to further establish the role of adiponectin. The study had specific exclusion criteria such as diabetes, prior revascularization, prior CAD, and renal failure. Hence the study sample is not representative of the general population of CAD patients at large. However, this was important to exclude the effect of these factors on the markers.

## 5. Conclusion

Present study suggested that higher plasma adiponectin levels are associated with a lower risk of ACS and possible lesser lesion severity, independent of other traditional metabolic and cardiovascular risk factors. These findings may have important implications both for understanding the pathophysiology of ACS and for the development of future therapeutic and coronary preventive approaches.

## Figures and Tables

**Table 1:** Baseline demographic and clinical characteristics of two groups.

Variables	Group I Non-ACS (*n* = 45)	Group II ACS (*n* = 45)	*P* value
Age (yrs) (mean ± SD)	57.49 ± 10.44	55.58 ± 12.10	0.42
Male, no. (%)	34 (75.6%)	35 (77.81%)	0.80
Female, no. (%)	11 (24.4%)	10 (22.2%)	0.80
BMI (Kg/m^2^) (mean ± SD)	23.82 ± 3.5	26.71 ± 4.09	0.001
Smoker, no. (%)	22 (48.9%)	28 (62.2%)	0.02
Hypertension, no. (%)	14 (31.1%)	23 (51.1%)	0.05
SBP (mm Hg) (mean ± SD)	127.9 ± 23.07	124.09 ± 23.29	0.43
DBP (mm Hg) (mean ± SD)	77.20 ± 15.35	76.53 ± 14.23	0.83
Family CAD history, no. (%)	9 (20%)	21 (46.7%)	0.007
Prior MI, no. (%)	18 (40%)	16 (35.6%)	0.66

ACS: acute coronary syndrome, BMI: basal metabolic index, SBP: systolic blood pressure, DBP: diastolic blood pressure, and MI: myocardial infarction.

**Table 2:** Biochemical variables of two groups.

Variables	Group I Non ACS (*n* = 45) (mean ± SD)	Group II ACS (*n* = 45) (mean ± SD)	*P* value
Glucose (mg/dL)	89.29 ± 14.19	87.51 ± 15.85	0.57
Insulin (*µ*u/mL)	21.15 ± 15.31	15.28 ± 8.57	0.09
Adiponectin (*µ*g/mL)	16.47 ± 7.88	9.03 ± 3.13	<0.001
Leptin (ng/mL)	9.26 ± 8.48	11.68 ± 8.26	0.037
hs CRP (mg/L)	3.78 ± 1.73	10.42 ± 4.97	<0.001
Triglycerides (mg/dL)	121.02 ± 58.6	139.76 ± 46.80	0.09
LDL cholesterol (mg/dL)	75.20 ± 32.41	83.91 ± 37.60	0.242
HDL cholesterol (mg/dL)	41.4 ± 7.73	39.16 ± 8.52	0.194
LDL/HDL ratio	1.85 ± 0.81	2.24 ± 1.078	0.057

ACS: acute coronary syndrome, hs CRP: high sensitive C reactive protein, LDL: low density lipoprotein, and HDL: high density lipoprotein.

**Table 3:** Angiographic profile of two groups.

Variables	Group I Non-ACS (*n* = 45)	Group II ACS (*n* = 45)	*P* valve
Significant CAD, no. (%)	40 (89%)	41 (91%)	0.56
Normal CAG, no. (%)	5 (11.1%)	4 (8.9%)	
SVD, no. (%)	20 (44.4%)	9 (20%)	
DVD, no. (%)	11 (24.4%)	14 (31.1%)	
TVD, no. (%)	9 (20%)	18 (40%)	

ACS: acute coronary syndrome, CAD: coronary artery disease, CAG: coronary angiography, SVD: single vessel disease, DVD: double vessel disease, and TVD: triple vessel disease.

**Table 4:** Correlations of adiponectin with other variables.

Variable	Correlation coefficient	*P* value
Age	0.060	0.572
BMI	−0.147	0.167
SBP	−0.011	0.921
DBP	−0.052	0.630
Triglyceride	−0.098	0.360
HDL cholesterol	0.321	0.002
LDL cholesterol	−0.207	0.051
LDL/HDL	−0.318	0.002
Blood glucose	0.081	0.450
Insulin levels	−0.016	0.883
hs CRP	−0.443	0.001
Leptin levels	−0.157	0.138

BMI: basal metabolic index, SBP: systolic blood pressure, DBP: diastolic blood pressure, hs CRP: high sensitive C reactive protein, LDL: Low density lipoprotein, and HDL: high density lipoprotein.

**Table 5:** Angiographic severity of coronary artery stenosis and adiponectin levels.

CAG- N/SVD/DVD/TVD	*N *	Mean adiponectin (*µ*g/mL)	Std. Deviation	*P* value
Normal	9	14.778	5.7820	0.023
SVD	29	16.031	9.2918	
DVD	25	10.644	4.8405	
TVD	27	10.833	5.1208	

CAG: coronary angiography, SVD: single vessel disease, DVD: double vessel disease, and TVD: triple vessel disease.
